# Heterogeneity of clinical symptomatology in pediatric patients at clinical high risk for psychosis

**DOI:** 10.1186/s13104-024-06742-7

**Published:** 2024-03-26

**Authors:** L. Sophia Rintell, Devon Carroll, Meghan Wales, Joseph Gonzalez-Heydrich, Eugene D’Angelo

**Affiliations:** 1 Department of Psychiatry and Behavioral Sciences, Boston Children’s Hospital, 300 Longwood Ave, 02115 Boston, MA USA; 2 Department of Psychology, Rosalind Franklin University of Medicine and Science, 3333 N Green Bay Rd., 60064 North Chicago, IL USA; 3 College of Nursing, University of Rhode Island, 350 Eddy St, 02903 Providence, RI USA; 4 Department of Psychiatry, Harvard Medical School, 401 Park Dr, 02215 Boston, MA USA

**Keywords:** Psychosis, Clinical high risk, Early onset psychosis, SIPS

## Abstract

**Objective:**

Widespread use of diagnostic tools like the Structured Interview for Prodromal Symptoms (SIPS) has highlighted that youth at Clinical High Risk for Psychosis (CHR-P) present with heterogeneous symptomatology. This pilot study aims to highlight the range of clinical characteristics of CHR-P youth, investigate the role of the non-positive (negative, disorganization, and general) symptoms in risk assessment, and determine if specific profiles are associated with severe symptomatology.

**Methods:**

38 participants aged 7–18 were administered the SIPS and designated as CHR-P. Descriptive statistics and mean difference t-tests were used to describe the range in prevalence and severity of SIPS symptoms and to identify symptoms associated with greater overall symptomatology.

**Results:**

Participants who had a greater number of positive symptoms also had significantly more negative, disorganization, and general symptoms. A number of SIPS symptoms were associated with greater number of positive symptoms.

**Conclusion:**

CHR-P youth represent a heterogeneous group, presenting with a wide range in clinical presentation as reflected in both the number of SIPS symptoms and their severity. Though the severity and duration of positive SIPS symptoms determines the CHR-P classification, high ratings on several of the other SIPS negative, disorganization, and general items may be useful indicators of elevated symptomatology.

## Introduction

There is a growing sense of urgency to investigate and better characterize the prodromal phase of psychosis, known as Clinical High Risk for Psychosis (CHR-P) in order to enhance early identification and prevention efforts [1]. Although it is typical for the onset of psychotic disorders to occur in young adulthood, Early Onset Psychosis (EOP; before age 18) accounts for one-third of cases of psychotic disorders [2]. Early detection of psychosis risk for children is critical since EOP leads to worse outcomes for children as compared to adults [3] and longer duration of untreated psychosis is also associated with worse outcomes [4]. Prospective attempts to identify individuals at-risk for psychosis have been guided by the development of semi-structured interviews to assess prodromal symptoms, the most common in the United States being the Structured Interview for Prodromal Symptoms (SIPS) [5]. The SIPS is intended to provide early identification of psychosis risk, yet work continues to be done to enhance its predictive accuracy for clinical outcomes for CHR-P individuals [6, 7]. For example, reported conversion rates in children at clinical high risk are inconsistent and modest in nature, ranging from 9.5 to 17.5% in the 12–72 months after identification [8–10]. Baseline symptomatic heterogeneity in the CHR-P population may be one explanation for the variability in clinical outcomes [11]. The SIPS, including the companion Scale of Prodromal Symptoms (SOPS) and Criteria of Psychosis Risk Syndromes (COPS), identifies three CHR-P risk syndromes: Genetic Risk and Deterioration Syndrome (GRD), Brief Limited Intermittent Psychotic Symptoms (BLIPS), and Attenuated Psychotic Symptoms (APS). In particular, GRD is found to be the least predictive of conversion, BLIPS is considered to be the most predictive of subsequent psychosis, and APS has mixed findings of conversion [12]. The most common risk syndrome, APS, accounts for 85% of CHR-P cases, and is determined based on the presence of one or more attenuated positive symptoms. These criteria are notably broad, with the potential to encompass individuals presenting with only one mild to five or more severe attenuated positive symptoms. Additionally, the SIPS also has the capacity to identify a wide range of clinical symptoms in addition to positive symptoms that at-risk individuals are known to exhibit, including negative, disorganization, and general symptoms [13]. Consequently, focusing exclusively on whether the APS classification criteria is met may overlook the diversity of the CHR-P patients’ overall symptoms and make it more challenging to understand the neurologic underpinnings of symptoms in patients with differing profiles.

The present pilot study (1) describes the heterogeneity of SIPS positive symptom presence and severity in CHR-P youth, (2) investigates the presence and role of the SIPS non-positive (negative, disorganization, and general) symptoms in risk assessment, and (3) compares symptom profiles of CHR-P youth who present with fewer positive attenuated symptoms versus those who have more positive attenuated symptoms, to determine if specific SIPS symptoms are associated with overall more severe symptomatology.

## Materials and methods

### Participants

In this pilot study, youth ages 7 to 18 were recruited from the outpatient service in a pediatric academic medical center in New England as part of a larger study of neuroplasticity and psychosis [14]. Potential participants were administered the Kiddie Schedule for Affective Disorders and Schizophrenia-Present and Lifetime version (KSADS-PL) [15] to rule-out the presence of a psychotic disorder and administered the SIPS to determine CHR-P status. Thirty-eight participants were enrolled on the basis of meeting criteria for at least one of the three CHR-P sub-groups. Exclusion criteria included the presence of a psychotic disorder as determined by the KSADS-PL and clinical chart review identifying substance-induced prodromal symptoms, intellectual disability, history of seizure disorders, and previous traumatic brain injury. Participants provided written informed assent or consent following the guidelines on ethical inclusion of children with psychosis in research described by Frost et al. [16], and all parents/guardians gave written informed consent. The project was approved by the academic institutional review board.

**Table 1:** Demographics

	Number (%)	Mean ± SD
**Gender**		
Female	19 (50)	
Male	17 (44.7)	
Transgender/non-binary	2 (5.3)	
**Age**		12.68 ± 2.82
7–13	21 (55.3)	
14–18	17 (44.7)	
**Race**		
White	28 (70)	
Black	2 (5)	
Asian American	3 (7.5)	
Bi/multi-racial	3 (7.5)	
Other	4 (10)	
**Ethnicity**		
Hispanic/Latino	7 (18.4)	
Non-Hispanic/Latino	29 (76.3)	
Missing	2 (5.3)	
**Income**		
$0–39,999	7 (18.4)	
$40,000–99,999	13 (34.2)	
$>100,000	12 (31.6)	
Missing	6 (15.8)	
**CHR category**		
APS	38 (100)	
GRD	3 (7.9)	
BIPS	0 (0)	
**Comorbid DSM diagnoses**		
Neurodevelopmental disorders	11 (28.9)	
Bipolar and related disorders	1 (2.6)	
Depressive disorders	18 (47.4)	
Anxiety disorders	15 (39.5)	
OCD and related disorders	1 (2.6)	
Trauma and stressor-related disorders	5 (13.2)	
Gender dysphoria	2 (5.3)	
Feeding and eating disorders	1 (2.6)	
Disruptive, impulse-control, and conduct disorders	5 (13.2)	
Personality disorders	1 (2.6)	
None	7 (18.4)	
Multiple	19 (50)	
**Missing**	1 (2.6)	
**N**	38	

### Measures

As previously noted, the Structured Interview for Prodromal Syndromes (SIPS) is a semi-structured interview that assesses psychotic-like symptoms in four categories: positive, negative, disorganization, and general symptoms. It contains a rating scale for individual symptom severity: The Scale of Prodromal Symptoms (SOPS), which ranges from 0 (absent) to 6 (severe and psychotic) and the Criteria of Psychosis Risk Syndromes (COPS), which determines the CHR-P risk syndrome. To meet criteria for CHR-P Attenuated Positive Symptom (APS) risk syndrome, an individual must receive a rating of 3 to 5 (indicating subthreshold severity) on at least one of the five positive symptoms that began or worsened within the last year and have been present in the last month [5]. The SIPS has demonstrated excellent sensitivity (100%), specificity (74%), and inter-rater reliability for all four subscales [5]. Trained research assistants administered the SIPS. Questions were further clarified and explained to any participant when necessary. Demographic data were obtained via a study specific demographics questionnaire in conjunction with a clinical chart review. Data analyses included descriptive statistics and mean difference t-tests to describe SIPS symptoms and identify symptoms associated with greater overall symptomatology.

## Results

Demographic information is summarized in Table 1. All participants met criteria for APS and three participants met criteria for both APS and Genetic Risk and Deterioration (GRD). No participants met criteria for the third psychosis risk syndrome, Brief Intermittent Psychosis (BIPS). Thirty-one (81.6%) participants met criteria for at least one other co-occurring DSM-5 disorder. Nineteen participants (50.0%) met criteria for multiple DSM-5 disorders. On average, participants endorsed 2.74 (SD = 1.13) attenuated positive symptoms (from a possible 1–5 symptoms). The number and severity of symptoms did not differ by gender or age.

The Mean Severity Rating, within a range from 1 (mild) to 6 (severe), for the five positive symptoms was 3.82 (SD = 0.77) across all participants. In addition to positive symptoms, participants presented with an average of 9.39 (SD = 3.28) (out of a possible 14) negative, disorganization, and general, symptoms with a mean severity rating per symptom of 1.74 (SD = 0.95) [within a range from 1 (mild) to 6 (severe)]. Table 2 provides frequencies for all of the SIPS symptoms. From the total list of SIPS symptoms, the most frequently reported symptoms were Trouble with Focus and Attention (D3) (*n* = 37; 97.4%), Perceptual Abnormalities/Hallucinations (P4) (*n* = 34; 89.5%), Dysphoric Mood (G2) (*n* = 34; 89.5%), and Avolition (N2) (*n* = 30; 78.9%).

**Table 2:** SIPS symptom frequency and severity ratings

Symptom	Frequency (%)	Severity rating mean ± SD
P1: Unusual thought content/delusional ideas	23 (60.5)	3.70 ± 0.76
P2: Suspiciousness/persecutory ideas	23 (60.5)	3.52 ± 0.79
P3: Grandiosity	4 (10.5)	3.50 ± 0.58
P4: Perceptual abnormalities/hallucinations	34 (89.5)	4.18 ± 0.72
P5: Disorganized communication	20 (52.6)	3.75 ± 0.72
N1: Social anhedonia	28 (73.7)	2.18 ± 1.54
N2: Avolition	30 (78.9)	3.27 ± 1.41
N3: Expression of emotion	20 (52.6)	2.35 ± 1.23
N4: Experience of emotions and self	28 (73.7)	2.50 ± 1.20
N5: Ideational richness	24 (63.2)	2.50 ± 1.18
N6: Occupational functioning	28 (73.7)	2.96 ± 1.26
D1: Odd behavior or appearance	14 (36.8)	2.57 ± 1.09
D2: Bizarre thinking	17 (44.7)	1.94 ± 1.03
D3: Trouble with focus and attention	37 (97.4)	2.65 ± 1.11
D4: Impairment in personal hygiene	18 (47.4)	2.00 ± 1.08
G1: Sleep disturbance	28 (93.3)	2.57 ± 0.96
G2: Dysphoric mood	34 (89.5)	3.03 ± 1.62
G3: Motor disturbances	18 (47.4)	2.00 ± 1.19
G4: Impaired tolerance to normal stress	29 (85.3)	2.79 ± 1.63
N	**38 (100)**	

In an effort to understand whether the severity of certain SIPS items was associated with increased psychotic-like symptomatology, participants were categorized into two groups based on the number of positive symptoms endorsed. These two groups were characterized by a higher number of attenuated positive symptoms (3–5) (High APS group) and a lower number of attenuated positive symptoms (1–2) (Low APS group). Independent samples t-tests were conducted to investigate differences in mean SIPS symptoms between the High APS group and the Low APS group. As seen in Table 3, there were significant differences in symptom severity between the High APS (*n* = 23; 60.53%) and the Low APS (*n* = 15; 39.47%) groups. The High APS group evidenced significantly higher ratings for Unusual Thought Content/Delusional Ideas (P1), Suspiciousness/Persecutory Ideas (P2), Disorganized Communication (P5), Avolition (N2), Occupational Functioning (N6), Bizarre Thinking (D2), Trouble with Focus and Attention (D3), Motor Disturbances (G3), and Impaired Tolerance to Normal Stress (G4). Table 3 includes t-test scores for all SIPS symptoms. Additionally, the High APS group exhibited significantly more negative symptoms (*M* = 4.70, *SD* = 1.69) than the Low APS group (*M* = 3.40, *SD* = 1.80), *t*(36) = -2.25, *p* =.031, more disorganization symptoms (*M* = 2.57, *SD* = 1.12) than the Low APS group (*M* = 1.80, *SD* = 1.01), *t*(36) = -2.13, *p* =.040, and more general symptoms (*M* = 3.35, *SD* = 0.83) than the Low APS group (*M* = 2.40, *SD* = 1.12), *t*(36) = -2.99, *p* =.005.

**Table 3:** Mean differences in SIPS symptom ratings

	Low APS group *n* = 15		High APS group *n* = 23		t-test
	M	SD	M	SD	
P1: Unusual thought content/delusional ideas	1.73	1.28	3.35	1.15	-4.04**
P2: Suspicious/persecutory ideas	1.33	1.23	3.09	1.47	-3.81**
P3: Grandiosity	0.33	0.62	0.96	1.40	-1.62
P4: Perceptual abnormalities/hallucinations	3.53	1.46	4.09	0.90	-1.45
P5: Disorganized communication	0.87	1.13	3.26	1.32	-5.78**
N1: Social anhedonia	1.13	1.19	1.91	1.83	-1.46
N2: Avolition	1.73	1.71	3.13	1.74	-2.44*
N3: Expression of emotion	0.73	1.16	1.57	1.59	-1.74
N4: Experience of emotions and self	1.53	1.46	2.04	1.55	-1.01
N5: Ideational richness	0.93	1.28	2.00	1.57	-2.20
N6: Occupational functioning	1.40	1.18	2.70	1.82	-2.44*
D1: Odd behavior or appearance	0.60	1.30	1.17	1.47	-1.23
D2: Bizarre thinking	0.33	0.72	1.22	1.31	-2.38*
D3: Trouble with focus and attention	2.07	1.03	2.91	1.16	-2.29*
D4: Impairment in personal hygiene	0.87	1.06	1.00	1.38	-0.32
G1: Sleep disturbance	1.40	1.35	2.22	1.38	-1.80
G2: Dysphoric mood	2.07	1.71	3.13	1.77	-1.84
G3: Motor disturbances	0.40	0.83	1.30	1.43	-2.22*
G4: Impaired tolerance to normal stress	1.67	1.50	2.91	1.83	-2.20*

*p <.05, **p <.001, df = 36.

## Discussion

As a pilot investigation, this study examined the nature of symptom heterogeneity in a subset of youth exhibiting clinical high risk for psychosis. The results suggest that there is a wide range in clinical presentation as reflected in both the number of SIPS positive symptoms endorsed and their severity. Our results suggest that, in addition to the positive symptoms, high ratings on several of the other SIPS negative, disorganization, and general items (e.g., Avolition, Occupational Functioning, Bizarre Thinking, Trouble with Focus and Attention, Motor Disturbances, and Impaired Tolerance to Normal Stress) may be useful indicators of elevated symptomatology.

The association between number of positive symptoms in the 3–5 range and the severity of attenuated positive symptoms as well as the negative, disorganization, and general SIPS symptoms, suggests the necessity of looking beyond simply the categorical assessment of CHR-P.

These findings provide support for the use of a dimensional conceptualization of psychosis [17]. van Os & Guloksuz [18] posit that given the SIPS already measures symptoms dimensionally (by presence and severity of symptoms), such a dimensional diagnostic method for CHR-P might be useful. The somewhat arbitrary nature of the categorical cut point used in APS criteria has been acknowledged as a limitation for the high-risk paradigm, however, has remained the predominant criteria used [19].

Awareness of the heterogeneity in CHR-P individuals, and youth in particular, has implications for treatment and prevention efforts. This study highlights that all youth at clinical high-risk for psychosis are not alike, have a wide range of presenting symptoms and functional outcomes, and may require distinct types of treatment depending on which symptoms are most prominent and impairing, regardless of actual rates of conversion. As such, it is prudent to discuss not only the SIPS positive symptoms but also negative, disorganization, and general symptoms in the context of clinical high risk.

A number of previous studies have used multivariate prediction models to investigate the predictive validity of the non-positive symptoms in risk for conversion to psychosis [20, 21]. Negative symptom severity [22–27], symptoms of disorganization [27–29], disordered thought content [30–32], and social dysfunction [33–35] have all been found to be predictive of conversion to psychosis. These findings indicate the possibility that non-positive symptoms may play a role in psychosis risk.

Consistent with the literature, the majority of CHR-P participants in this study had one or more co-occurring DSM-5 diagnoses, which confirms that most CHR-P individuals are diagnosed with common mental disorders that may persist over time [36, 37], a finding which is consistent for children and adolescents in particular [38]. It is crucial to acknowledge this comorbidity in order to provide specific treatments that are indicated for individuals who have psychotic experiences with co-occurring psychopathology. Moreover, given the heterogeneity of the symptoms evidenced by the participants in this study, additional research on the impact of comorbid disorders on conversion risk is needed, as well as research on the efficacy of treatments for psychosis with specific comorbidities.

## Limitations

This pilot study should be interpreted cautiously since it contains several limitations. First, the small sample size and limited demographics, such as predominantly middle to upper class white and non-Hispanic/Latino participants, may adversely affect our ability to more broadly describe the true range in symptom heterogeneity in CHR-P youth. Second, this sample represents only help-seeking individuals. Third, because this was a pilot study, follow-up clinical outcome data such as conversion to psychosis was not collected from the participants. It may not be the case that the presence of more positive symptoms is a marker for increased risk of conversion to psychosis.

## Conclusion

While identification of factors that contribute to the risk for conversion to psychosis is needed, it is important to acknowledge that even those who do not convert face challenges and remain vulnerable, with only half remitting over time [23, 39]. Individuals at CHR-P are not only likely to present with persistent comorbid diagnoses, but are likely to experience the onset of additional disorders [37], and often experience persistent functional impairment and attenuated psychosis [40, 41]. In general, CHR-P individuals are likely to experience reduced quality of life [42] and elevated levels of stress, reiterating the need for comprehensive assessment and treatment in this population [43]. It is increasingly apparent that CHR-P youth represent an extremely vulnerable population with heterogeneous clinical presentations. A greater understanding of their clinical profiles will aid in preventing conversion and poor outcomes for these individuals.

## Data Availability

The dataset that supports the findings of this study is not publicly available due to its containing identifiable information that could compromise the privacy of research participants but is potentially available from the corresponding author [E.D.] upon reasonable request.
